# “Multi-Omics” Analyses of the Development and Function of Natural Killer Cells

**DOI:** 10.3389/fimmu.2017.01095

**Published:** 2017-09-05

**Authors:** Yonggang Zhou, Xiuxiu Xu, Zhigang Tian, Haiming Wei

**Affiliations:** ^1^School of Life Science and Medical Center, Institute of Immunology, CAS Key Laboratory of Innate Immunity and Chronic Disease, University of Science and Technology of China, Hefei, China; ^2^Hefei National Laboratory for Physical Sciences at Microscale, University of Science and Technology of China, Hefei, China

**Keywords:** “omics” technology, “multi-omics”, natural killer cell molecular program, natural killer cell diversity, natural killer cell immunotherapy

## Abstract

For over four decades, our understanding of natural killer (NK) cells has evolved from the original description of cluster of differentiation (CD)56^+^CD3^−^ to establishing NK cells as an important subset of innate lymphocytes in the host’s surveillance against viral infections and malignancy. The progress of research on the fundamental properties and therapeutic prospects for translational medicine using NK cells excites immunologists and clinicians. Over the past decade, numerous advances in “-omics”-scale methods and new technological approaches have addressed many essential questions in the biology of NK cells. We now have further understanding of the overall molecular mechanisms of action that determine the development, function, plasticity, diversity, and immune reactivity of NK cells. These findings are summarized here, and our view on how to study NK cells using “multi-omics” is highlighted. We also describe “-omics” analyses of the relationships between NK cells and viral infection, tumorigenesis, and autoimmune diseases. Ultimately, a deeper and more comprehensive understanding of NK cells in multiple conditions will provide more effective strategies to manipulate NK cells for the treatment of human disease.

## Introduction

As early as 1975, in some experiments carried out *in vitro*, a phenomenon was noticed: some lymphocytes of an undefined type from the normal mouse spleen selectively fought against Moloney leukemia cells spontaneously (1). In 1979, the same cell functions were also described in healthy humans (2, 3). The phenomenon was described as “natural cytotoxicity,” and the lymphocytes were ultimately named “natural killer” (NK) cells.

Initially, NK cells were believed to act as just an “annoying” background of cytolytic activity in several cell lineages. In 1986, as a result of the discovery of several cell surface markers and the confirmation of natural cytotoxicity, NK cells were determined to be a new lineage of lymphocytes (4, 5). The first 30 years of research into NK cells were focused mainly on descriptions of the functions and the identification of single surface markers. The overall progress into research of NK cells was slow and lagged behind that of most other types of immune cells.

Over the past decade, developments in “-omics”-scale technology, such as analyses of gene expression as well as quantification of proteins and metabolites, have enriched our understanding of the complex biologic processes of NK cells. This understanding includes their phylogeny, developmental programs, plasticity, and immune reactivity for controlling viral infections and malignancy at the molecular level. More importantly, NK cells have recently attracted attention for their therapeutic prospects in cellular immunotherapy due to technical progress that has helped immunologists and clinicians gain a better understanding of NK cells (6).

The English-language neologism “-omics” contains several specific molecular levels, such as “genomics” (the sequence and expression of DNA), “transcriptomics” (DNA transcription into RNA), “proteomics” (RNA translation into proteins), or “metabolomics” (metabolites). These methods generate large data sets, which are often referred to as “-omics” data. The expansion in “-omics” methods is due to mainly tremendous advancements in technology through approaches such as next-generation sequencing (NGS) and mass spectrometry (MS).

**Figure 1:**
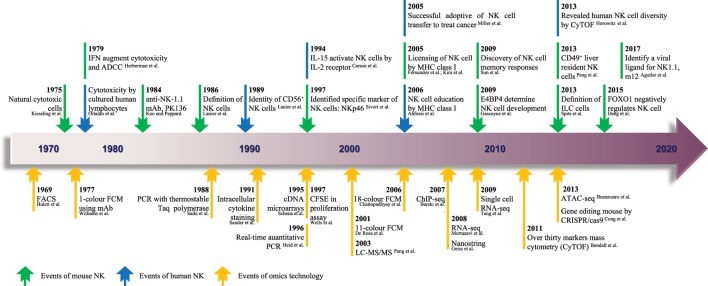
The timeline of NK cell research keeps pace with advances in “-omics” technology. Since the identification of NK cells in 1975, analytical methods for NK cells were based mainly on FCM and gene expression analyses. From 1969 to the present day, rapid technological advances in FCM and gene expression analyses have become high-throughput technologies in the true sense, and there is much hope for the future. The top row shows the timeline of events related to NK cell research. Blue represents NK cells in general, and green denotes mouse NK cells. The bottom row shows the progress of events related to “-omics” technology. NK, natural killer; ADCC, antibody-dependent cell-mediated cytotoxicity; mAb, monoclonal antibody; ILC, innate lymphoid cell; FACS, fluorescence-activated cell sorting; FCM, flow cytometry; PCR, polymerase chain reaction; CFSE, carboxyfluorescein succinimidyl ester; ChIP-seq, chromatin immunoprecipitation sequencing; RNA-seq, RNA sequencing; ATAC-seq, assay for transposase-accessible chromatin sequencing; CRISPR, clustered regularly interspaced short palindromic repeats; LC-MS/MS, liquid chromatography–tandem mass spectrometry. The references cited in this figure are all listed in the data sheet (Supplementary Material).

In this review, we highlight the “-omics”-scale data that have assisted research of NK cells (Figure 1), including methods that examine their phenotypes, transcriptional signatures, and effector functions in various biologic processes or *niches*. This approach can help to constantly update the “road map” of gene expression that forms a more comprehensive regulation network and provides new strategies to manipulate NK cells for the treatment of human disease.

## “Omics” Technology Applied to the Study of NK Cells

Aside from metabolomics, most “-omics” technologies are usually considered to be based on genomics, which arose largely from the deciphering of the complete human genome (7, 8) and mouse genome (9) in the early 2000s, undertakings that marked a new milestone in the life sciences. “Omics” technology presents a panoramic view of the unbiased molecular determinants of NK cells not only from development to an exhaustive process but also for multiple responses to effector function in different environments. To analyze NK cells using different levels of “-omics” approaches, very different biotechnologies are applied in each case.

### Microarrays Technology

Due to the increasing efficiency of chips (10) and the constantly increasing number of available monoclonal antibodies, thousands of biologic reactions at DNA, RNA, or protein levels can be measured or even quantified (11) in a single experiment. As the earliest high-throughput method to analyze gene transcription and protein expression, microarray technology (12) (Figure 1) has made significant contributions to the rapid development of research of NK cells over the last decade. Although NK cells have been considered for a long time to be natural soldiers against viral infection and cancer in the body, the key transcription factors (TFs) that regulate the responses of NK cells to viral infection are poorly understood.

Therefore, to screen key TFs, researchers used microarray technology to compare and analyze changes in gene expression in purified Ly49H^+^ NK cells from murine cytomegalovirus (MCMV)-infected and control mice (13) (Table 1). In this experiment alone, >30,000 genes were evaluated on a microarray, and *Zbtb32* was screened because it was one of the most highly upregulated genes after MCMV infection. These data were confirmed through quantitative reverse transcription-polymerase chain reaction. This experiment is a classic instance of how to screen key genes in an important biologic process by microarray analysis. In addition, microarray technology is also used widely for studying the phenotypic and functional molecular signatures of NK cells. Wang and colleagues, using sorted populations of human NK cells from decidual, cord blood, and peripheral blood, investigated novel phenotypic and functional molecular signatures and transcriptional regulators by whole-genome microarray analysis (14) (Table 1). Through a comparative analysis of gene profiles of NK cells from those sources, the authors highlighted the differences in surface receptors, chemokine receptors, TFs, and functional molecules of NK cell populations. Interestingly, that research indicated that decidual natural killer (dNK) cells may specifically express some new growth factors, cytokines, and chemokine genes; the identification of these genes is helpful for the functional classification of dNK cells. More notably, they showed that TF expression in dNK cells and peripheral natural killer (pNK) cells has family preferences: dNK cells are enriched for the homeobox family, whereas pNK cells express zinc-finger family TFs predominantly. The two studies mentioned above have been cited extensively by other researchers in cell biology.

**Table 1:** Application of “Omics technologies” in complex NK cell research.

Species	Sample	Method	Keypoint	Reference
**Transcriptomics: microarray related studies in NK cells (mRNA/miRNA/LncRNA)**				
Mo	NK. Sp./Lv./SI.	P: Affymetrix MoGene 1.0 ST array	1. ILC1. Lv.: CD49a^+^, TRAIL^+^	GSE37448 (15)
			2. ILC1. SP.: CD127^+^, Eomes^−^	
			3. ILC1. SI.: CD27^−^, Eomes^−^	
	ILC1. Sp./Lv./SI.	A: GenePattern; PCA		
Mo	NK. Sp. Healthy	P: Affymetrix MoGene 1.0 ST array	*Zbtb32* controls expansion of virus-specific NK	GSE15907 (13)
	NK. Sp. MCMV	A: GenePattern		
Hu	NK. PB./CB./D.	P: Whole HuGenome Oligo Microarray	1. Homeobox TFs enrich in dNK	GSE24268 (14)
		A: Agilent’s Feature-Extraction v 9.1.3	2. Zinc-finger TFs enrich in pNK;	
Hu	NK. PB./CB./D.	P: Hu miRNA microarray	1. Inhibitory miRNA: miR-483-3p	GSE66325 (16, 17)
		A: Agilent’s Feature-Extraction v 9.5.3.1	2. Activated miRNA: miR-362-5p	
Hu	NK. PB./CB./D.	P: Agilent Hu180K lncRNA and mRNA microarray	Lnc-CD56 upregulates CD56	(18)

**Transcriptomics: mRNA-seq-related studies in NK cells**				
Mo	CD49a^+^ NK. Lv./Sp./BM.	P: HiSeq 2500	1. trNK: CD49a^+^, CD69^+^	(19)
	DX5^+^ NK. Lv./Sp./BM.	A: ESAT software	2. trNK is depend on T-bet	

**Transcriptomics: scRNA-seq-related studies in NK cells**				
Hu	ILCs. Tn	L: SMART-seq2 Pro.	1. Human ILCs express *RARG*	(20)
		P: HiSeq2000		
	NK. Tn	A: STAR v2.3.0, SCDE	2. Mature ILCs including NK cells express PLZF, unlike mice	
Mo	WT. CLP.	L: SMART-seq2 Pro.	PD-1^+^ ILCP	(21)
	*Bcl11b*^−/−^. CLP.	P: HiSeq2000; A: DESeq2, SPADE		

**Transcriptomics: miRNA-seq-related studies in NK cells**				
Mo	NK. Sp. Resting	P: GA (Illumina) seq; SOLiD seq	Inhibitory miRNA: miR-223	GSE21003 (22)
	NK. Sp. IL-15-activated	A: pipeline v 0.2.2, SHRiMP		

**Transcriptomics: ATAC-Seq related studies in NK cells**				
Mo	NK. Sp./Lv.	ATAC-Seq, P: HiSeq 2500	Regulomes of ILCs VS T cells:	GSE77695 (23)
	ILC1. Lv.	A: MACS v 1.4.2, HOMER v 4.8		
	HSC. BM.	ChIP-Seq, P: HiSeq 2500	1. The regulator of the ILC effector genes is easier to open	
	CLP. BM.	A: SICER, MACS v 1.4.2		
	NKp. BM.	RNA-seq, P: HiSeq 2000	2. Regulomes of ILCs arborize early at precursor stages	
	imNK. BM.	A: Cufflinks 2.2.1		

**Proteomic: CyTOF-related studies in NK cells**				
Hu	NK. CB./PB. Healthy	P: Mass cytometer (Fluidigm)	The increased diversity of NK cells affects the function	(24)
	NK. PB. HIV	A: Inverse Simpson Index		
Hu	NK. PB.	P: Mass cytometer (Fluidigm)	CD49e^−^ trNK in human liver	(25)
	NK. L-PxF.	A: SPADE; Cytobank		

**Proteomic: LC-MS/MS-related studies in hematopoietic cells (focused on NK cells)**				
Hu	CD56^bright^ NK. PB.	P: UHPLC, Q Exactive HF	The effect genes of NK and T_EM_ cells are similar	PXD004352 (26)
	CD56^dim^ NK. PB.	A: MaxQuant v1.5.3.2, Communication		

**CRISPR-related studies in NK cells**				
Mo	*SFRs*^−/−^ NK.	P: CRISPR	SFRs for NK cell education	(27)
		A: Sequencing; FACS		

*Mo, mouse; Hu, Human; Sp, spleen; Lv, Liver; BM, bone marrow; SI, small intestinal lamina propria; L-PxF, liver postexcision flush; PB, peripheral blood mononuclear cell; CB, cord blood mononuclear cells; D, decidual mononuclear cells; Tn, tonsil; SFRs, signaling lymphocytic activation molecule family receptors library preparation; TFs, transcription factors; HSCs, hemopoietic stem cells; CLP, common lymphoid progenitors; trNK, tissue-resident natural killer; NKp, natural killer cell precursor; imNK, immature natural killer; cNK, conventional natural killer; ILCP, precursors of innate lymphoid cells; L, library preparation; P, platform; A, analysis; Pro., protocol; scRNA-seq, single-cell RNA sequencing; ATAC-Seq, assay for transposase-accessible chromatin sequencing; ChIP-seq, chromatin immunoprecipitation sequencing; CyTOF, cytometry by time of flight; LC-MS/MS, liquid chromatography–tandem mass spectrometry; CRISPR, clustered regularly interspaced short palindromic repeats; SCDE, single-cell differential expression; SPADE, spanning-tree progression analysis of density-normalized events; SICER, Spatial clustering for identification of ChIP-enriched regions; UHPLC, ultra-high performance liquid chromatography; CD, cluster of differentiation; dNK, decidual natural killer; pNK, peripheral natural killer; RNA-seq, RNA sequencing; ILC, innate lymphoid cell; IL, interleukin; ncRNA, non-coding RNA; FACS, fluorescence-activated cell sorting; HIV, human immunodeficiency virus; MCMV, murine cytomegalovirus*.

Based on microarray technology, immunologists and computational biologists proposed the Immunological Genome Project (ImmGen), which is currently building a gene expression database for all characterized immune cells in the mouse (28). All data generated as a part of ImmGen are available freely and publicly on www.immgen.org.

### RNA Sequencing (RNA-Seq)

At the height of use of microarray technology, researchers intending to study gene profiles used gene arrays. However, in 2005, Solexa technology (Illumina) and SOLiD technology (Life Technologies) emerged as key symbols in the evolution of NGS. As the cost of sequencing plummeted, RNA-seq became an increasingly popular method of transcriptome analysis. Unlike microarray technology (which relies on fluorescent labeling), RNA-seq mainly transforms RNA into a cDNA library, which is followed by direct sequencing (29) (Figure 1). Under the condition of sufficient sequencing depth, RNA-seq is applied to analyze the differential elements of gene expression of the whole transcriptome in a more accurate, reproducible, wider, and more reliable manner than that of other methods (30, 31).

In addition to analyzing the levels of gene expression, RNA-seq can also identify new transcripts and splice variants and can measure allele-specific gene expression. Therefore, RNA-seq applied to analyses of the gene expression profile in these areas has more advantages than that of microarray analysis. RNA-seq has many advantages, but several researchers continue to use chips, especially if the sample size is large. Because its data processing is fast and simple and the raw RNA data are troublesome, bioinformatists are required to adopt different strategies of data analysis based on the design and target of the experiment. Currently, some RNA-seq data analysis programs have been published and some professional analytical software has been updated constantly.

RNA sequencing has also been applied in the research of NK cells. Since cluster of differentiation (CD)49a^+^ DX5^−^ was identified as the iconic marker of tissue-resident natural killer (trNK) cells in the liver (32), research on trNK cells has moved rapidly. To characterize the molecular profile of trNK cells in the liver more precisely, RNA-seq was used to analyze purified CD49a^+^ DX5^−^ and CD49a^−^ DX5^+^, which are two subsets of NK cells from the liver, spleen, and bone marrow of mice (19) (Table 1). Results showed that trNK cells in the liver are a unique lineage of mature NK cells that are different from several reported NK cell subsets (19). By contrast, a basic hierarchical clustering analysis among different populations revealed that liver trNK cells displayed DX5^−^CD49a^+^ CD69^+^ CD44^+^ CD160^+^-specific signatures and were depend on T-box expressed in T cells (T-bet) and not nuclear factor, interleukin 3 regulated (NFIL3) (19) (Table 1). Wang et al. and Sojka et al. showed that microarray and RNA-seq technologies were useful to analyze the differences between NK cell subsets and for a comprehensive assessment of new subsets. However, better performance, lower costs, and help from bioinformatics have led RNA-seq to be favored. Furthermore, RNA-seq technology is also improving to help solve more complex problems.

### Single-Cell RNA Sequencing (scRNA-Seq)

There is now a general consensus that cell heterogeneity is common and normal. Whether microarray or RNA-seq technology need to extract a bulk RNA from more than 10^5^ cells, and the data obtained are the average values of cell populations (33). These methods cannot meet the demand of immunologists to study the diversity of immune cells, and even some important information may be ignored.

Recently, with technological advances in the separation of single cells and the establishment of cDNA libraries, scRNA-seq technology has emerged to make it easier to analyze the molecular profile of the single cell from cell populations (34) (Figure 1). Innate lymphoid cells (ILCs), including helper-like cells (ILC1, ILC2, and ILC3) and conventional natural killer (cNK) cells (35), are a new paradigm of immune cells that mirror the helper T cell subsets that produce similar functional molecules (36, 37). ScRNA-seq as an important technological advance of RNA-seq that can provide great opportunities for ILCs research. As a highly heterogeneous cell population and with a restriction of cell numbers, studying the developmental trajectory and signatures of ILC progenitor cells is a problem. To address this problem, researchers delineated distinct ILC development stages and reported that PD-1^hi^ could be used as a marker of ILC precursor cells by undertaking scRNA-seq of bone marrow progenitor cells (21) (Table 1). The identification of PD-1^hi^ ILC precursor cells had a positive effect on tumor immunotherapy of PD-1 antibody (21). As research has progressed, scholars have now identified a brand new level of complexity in biology.

### MicroRNA Sequencing (miRNA-Seq)

miRNA molecules, although small, are powerful regulators of gene expression, and they are also expected to be markers of the diagnosis and therapeutic targets of a particular disease. Most of the research on the miRNA of NK cells can be done through microarray technology. This approach has led to the discovery of the inhibitory miRNA miR-483-3p (16) and the activated miRNA miR-362-5p (17) in human NK cells (Table 1).

However, if microarray technology and miRNA-seq are compared, the latter may have some obvious advantages. miRNA-seq can overcome the limitations of microarray technology (which is reliant on known miRNAs) to identify new miRNAs. miRNA-seq can even detect the difference in a single base of miRNAs. To improve the detection resolution and screen new miRNAs that regulate the function of NK cells, miRNA-seq was used to analyze the changes of miRNAs across the whole transcriptome during the activation of NK cells in mouse spleens by interleukin (IL)-15 (22) (Table 1). The library of this project was completed through two sequencing platforms: GA (Illumina) and SOLiD. Although there were small differences between the results of the two sequencing platforms, some new miRNAs were identified, and miR-223 was found to be an important regulator that inhibited the activation of NK cells (22).

### Assay for Transposase-Accessible Chromatin Sequencing (ATAC-Seq)

Similar to miRNAs, TFs are important elements of gene expression. However, TF functions are dependent on the specific and accessible chromatin regions in the genome. Currently, the most common methods used for the identification of accessible chromatin regions are chromatin immunoprecipitation sequencing (ChIP-seq) (38) and ATAC-seq (39) (Figure 1).

Chromatin immunoprecipitation sequencing can directly detect DNA sequences that bind to TFs, but a single sequencing can only provide information about a definitive TF (38). ATAC-seq requires only a few cells and simple experimental steps, and, after sequencing, all the accessible chromatin regions of chromosomes at a particular time and space can be obtained, and these are not confined to a TF-binding site or a specific area of histone acetylation (40). As mentioned above, the study of the transcriptional regulatory elements within a cell is essential for a comprehensive understanding of how the cell operates. To conduct a panoramic study on the transcriptional regulatory elements of ILCs, Shih and colleagues used ATAC-seq to analyze all of the prototypical subsets of ILCs from mice, including cNK cells (23) (Table 1). Focusing on the regulatory elements of the functional genes of ILCs, they showed that ILCs and T cells expressed similar functional genes to resist infection, but that the gene-regulatory elements of ILCs were more likely to be activated (23).

### Mass Cytometry

Compared with genes, proteins are the main components of life activities. Thus, biologists have been eager to implement high-throughput detection of cellular proteins. Flow cytometry (FCM) based on antibodies coupled with fluorescent compounds is the most common method used to analyze the proteins expressed on cells (41). To avoid overlap between wavelengths, the number of samples researchers can process is limited, so this method cannot achieve high-resolution detection.

Recently, a novel technology termed mass cytometry also known as cytometry with time-of-flight mass spectrometry (CyTOF) (42) (Figure 1) was developed. This merging of FCM and MS was developed to provide measurements of >40 coinstantaneous cellular parameters at single-cell resolution, and it has enhanced the detection ability considerably to explore complicated cellular systems (43). CyTOF allows for single-cell analysis of a larger number of markers than conventional FCM. A study by Bendall and colleagues demonstrated the applications of this technology for the first time using hematopoiesis, and the data were analyzed by spanning-tree progression analysis of density-normalized events (SPADE) (42). Then, the technology was demonstrated by applying the use of human leukocyte antigen (HLA) class-I tetramers to identify and model antigen-specific T cells (44).

By using CyTOF to analyze the expression of human pNK cell receptors in five sets of monozygotic twins, Horowitz and Blish described an unexpected scale of NK cell diversity and provided valuable evidence for an unsubstantiated hypothesis that genetic factors can control the expression of inhibitory receptors, whereas environmental factors may alter the expression of activated receptors (45, 46) (Table 1). Whereafter, by using CyTOF to study CMV reactivation in transplantation settings for acute myeloid leukemia (AML), Horowitz and colleagues discovered strong associations with HLA-C upregulation and increased expression of inhibitory killer cell immunoglobulin-like receptor (KIR) on effector memory CD8 T cells (47). CyTOF has also been used to measure cytokine-induced memory-like NK cells that were expected to be used in AML therapy by Todd Fehniger’s group (48). Another study by Blish and colleagues on NK cell diversity associated with antiviral function made good use of CyTOF (24).

Although there have been some reports of MS being used for immunologic studies, conventional FCM based on fluorescence continues to dominate and maintain a valuable role in the immunologist’s toolbox. This problem could be because (i) MS is limited by slow detection speed so a large number of samples cannot be detected; (ii) unique requirements for antibody labeling lead to the price of an individual panel being higher; and (iii) a method to sort and purify the detected cell population of interest is not available. Nevertheless, we believe that the continuous improvement and wider application of CyTOF will provide more useful data to immunologists with regard to complex subsets of immune cells.

### Liquid Chromatography–Tandem Mass Spectrometry (LC-MS/MS)

Cytometry by time of flight can detect the expression of >30 proteins in a single sample, but the extremely complex proteomics of cells cannot be evaluated. Nevertheless, a panoramic image of the cell proteome is needed urgently (26).

LC-MS/MS (i.e., a LC separator combined with a tandem MS detector) is a versatile, highly accurate, highly sensitive, and automated method for the qualitative and quantitative analyses of most small molecules. LC-MS/MS was first used for the study of yeast proteomics in 2003 (49) (Figure 1). Rieckmann and colleagues demonstrated a new, complex, and comprehensive research project of 28 hematopoietic cell types by single-shot LC-MS/MS (26) (http://www.immprot.org/). By recording the differences, clustering, and principal component analysis of different cells, they showed that, based on the relationship between functional proteins, a complex “social network” can be formed among immune cells and that the nearest partner of NK cells are CD8^+^ T effector memory cells (26) (Table 1). These findings are similar to the results of ATAC-seq described above, and both sets of findings were reported using big data analyses to show that NK cells have the same antiinfection function as adaptive immune cells.

### Gene Knockout with Clustered Regularly Interspaced Short Palindromic Repeats (CRISPR)

Immunologists are keen to confirm the functions of genes or proteins. Gene knockout mice have long been considered the gold standard for functional analyses *in vivo*. CRISPR/Cas9 is a newly developed gene-editing technology (50, 51) (Figure 1). CRISPR/Cas9 is very exciting because it not only greatly improves the efficiency of gene knockout in mice (52, 53) but also makes it possible to construct mice with simultaneous knockouts in multiple genes. Chen and colleagues used CRISPR/Cas9 genome-editing technology, and, through the distribution of multi-point gene targeting, they knocked-out 10 genes in signaling lymphocytic activation molecule (SLAM) receptors and SLAM-associated protein family proteins in mice. These animals revealed a new mechanism of acquisition of NK cell function and solved the issue of “SLAM family receptor redundancy,” which has been recognized as a problem in this research field (27) (Table 1). Biologists around the world are riding a wave of new technologies made possible by CRISPR.

From qPCR to microarray, from RNA-seq to scRNA-seq, and from traditional FCM to MS, “-omics” technologies are undergoing rapid development and will continue to be updated and enriched. These “-omics” technologies are facilitating a revolution in the research of the developmental and functional analyses of immune cells. The massive amounts of data generated using these methods are critical for understanding the contributions of immune cells to disease prevention and also for taking advantage of their full potential in immune cell-based therapies.

## “Omics” Technologies Power Furthering Understanding of NK Cells

**Figure 2:**
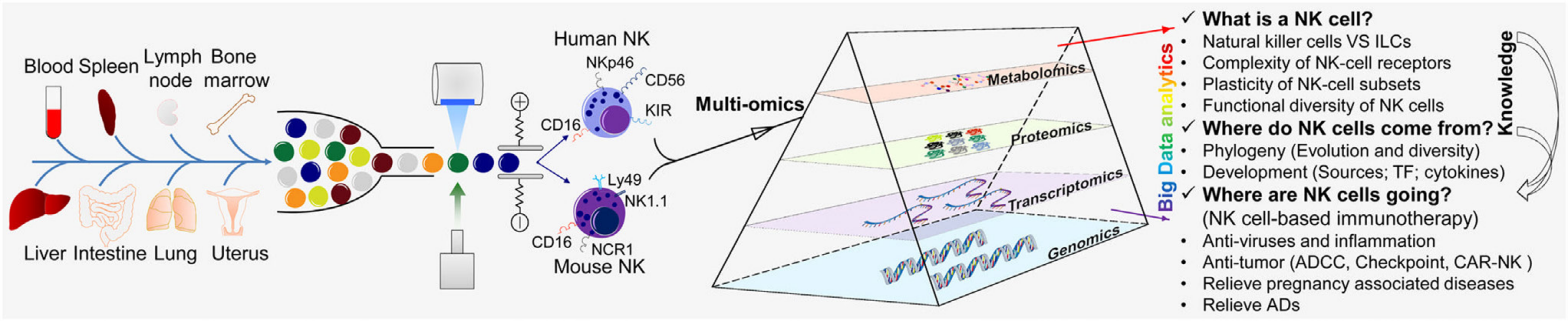
“Multi-omics” analyses for the further understanding of NK cells. “Multi-omics” analytical design and key questions to be addressed for NK cells (schematic). NK cells are an important part of the first line of defense for the body. NK cells are present in most of the tissues and organs of humans and mice, including blood, spleen, lymph nodes, bone marrow, liver, lungs, and uterus. NK cells were isolated by flow cytometry and analyzed by various “-omics” technologies in steady and activated states. Just like a prism refracting the seven colors comprising white light, “-omics” and “multi-omics” analyses of the intricate critical problems of NK cells can produce massive amounts of data and a panoramic view by sequencing, mass spectrometry, and LC-MS/MS and could help to solve these problems. There are three major research aspects of NK cells: (i) understanding NK cells from multiple perspectives (“what is a NK cell?”); (ii) the origin and development of NK cells (“where do NK cells come from?”); and (iii) how NK cells may be transformed and applied (“where are NK cells going?”). The knowledge provided by basic research can guide and serve the clinical transformation of NK cells. TF, transcription factor; ADCC, antibody-dependent cell-mediated cytotoxicity; CAR-NK, chimeric antigen receptor-engineered natural killer cell; NK, natural killer cell; ILC, innate lymphoid cell.

Thanks to the tireless efforts of immunologists, the study of NK cells has made great progress, and we have a more extensive understanding of NK cells. However, due to their complexity, three major research questions regarding NK cells remain: (i) understanding of NK cells from multiple perspectives (what is a NK cell?); (ii) the origin and development of NK cells (where do NK cells come from?); and whether NK cells can be transformed and applied (where are NK cells going?) (Figure 2).

These problems are complicated, but they can be divided into different levels and solved using a single “-omics” or a combination of multiple “-omics” (“multi-omics”) (Figure 2). “Omics” analyses are based on selecting the “-omics” technology and making the corresponding programs according to the experimental target.

First, in the study of the characteristics of NK cells, molecular profiles (e.g., transcriptome, proteome) were often analyzed comparatively among different cell types (e.g., NK cell subsets, NK cells, and ILCs) by microarray (14), RNA-seq (19), and CyTOF (25) (Table 1). Those studies revealed the unique profiles of gene expression or protein expression of different types of NK cells, but did not detail the key molecular mechanisms or carry out integration of data analyses to identify new regulatory elements. Furthermore, the “multi-omics” analysis of RNA-seq and mass spectrometric can also greatly improve the reliability of data and compensate for the shortage of data repeatability of small samples. But in terms of the project by Rieckmann and colleagues, it contains only the most classic human NK cell subsets: CD56^bright^ and CD56^dim^, although it is the credible and ambitious resource (26). In addition, the diversity of NK cell receptors has been a problem for researchers (54), especially the KIR (human) or Ly49 (mouse) families. “Multi-omics” could be used to study their regulomes by combining ATAC-seq with RNA-seq (23).

Second, innate immunity is a protective mechanism present in many types in plants and animals and even in prokaryotes (5, 55). The phylogeny of NK cells is not well understood. NK-like cells and some important receptor families related to the receptors of NK cells in mammals have been confirmed in bony fishes, amphibians, reptiles, and birds (56–58). In addition, with the rapid development of NGS technology and after mapping of the human and mouse genomes, a great deal of species-level genome sequencing has been completed. These big data sets of genomics could help decipher the phylogeny of NK cells according to the cluster of characteristic genes that contain multiple aspects of NK cells.

Third, in the study of disease-related NK cells, “multi-omics” that contain transcriptome, proteome, and even metabolome is an effective means of research (Figure 2). Paired single-cell analyses by scRNA-seq, RNA-seq, and CyTOF in combination have been used to describe the immune environment in lung cancer tissues and showed that the number of NK cells is severely reduced and impaired during the progression of lung cancer (59). It has been suggested that tumor immunotherapy of NK cells may be effective only in the early stage of lung cancer, but a new therapeutic target or possible methods are lacking.

## Dissecting the Whole Transcriptome Network of NK Cell Development

From NFIL3, the first relatively specific TF (60, 61) reported, to Forkhead box protein O1 (FOXO1), the first negative TF (62) reported, the past decade has seen a sharp increase in research of the transcriptional regulation of NK cell development.

Nuclear factor, interleukin 3 regulated is a crucial regulator for the early development of NK cells and commitment to the NK lineage because *Nfil3^−/−^* mice exhibit impaired production of NK cells at the transition of NK precursor cells to immature NK cells in the bone marrow (60). NFIL3 acts in the positive feedback loop of the IL-15 receptor (CD122) (63) by determining the expression of the downstream TFs Id2 and eomesodermin (EOMES) directly (60, 64, 65). Although several TFs have roles in NK cell development, not only Eomes but also T-bet regulate the development and function of NK cells (66). T-bet is known to be the critical TF of interferon (IFN)-γ production downstream of the IL-12 pathway and drives the development of T-helper 1 cells (67). With regarding to NK cells in the bone marrow, *Tbx21^−/−^* mice can block the production of NK cells at the transition from stage III (CD27^+^CD11b^+^) to stage IV (CD27^−^CD11b^+^) (68). Many target genes of T-bet and EOMES necessary for the appropriate development of NK cells and selective regulation of effector functions have been identified, such as *Ifn-*γ, *Granzyme B, Perforin, Blimp1*, and *S1p5* (68–70). T-bet and EOMES synergize the transcriptional regulation of cytotoxic factors in NK cells (66). Because T-bet is so important, several recent studies have focused on the negative factors or checkpoints for T-bet. FOXO1 downregulates T-bet expression (62) or mothers against decapentaplegic homolog 3 (SMAD3) downregulates NFIL3 expression (71) to impair the maturation and function of NK cells. Although those studies have used various “-omics” technologies and gene knockout mice, they have not described the entire transcriptional regulatory network of NK cell development due to a lack of research on posttranscriptional regulation.

It is also becoming evident that the development and functions of NK cells are not only regulated by TFs but are also influenced by posttranscriptional regulation through non-coding RNAs (ncRNAs) (72). Recent studies have shown that ncRNAs, including miRNAs, that are short ncRNAs (19–26 nt) and long ncRNAs (>200 nt), are also important for the development and function of NK cells (73, 74). Microarray analyses have been used to screen miRNAs in different NK cells from different tissues and shown that miR-483-3p decreases the cytotoxicity of NK cells due to inhibition of activated signal transducer and activator of transcription 5 by insulin-like growth factor 1 (16). Studies have also shown that miR-362-5p facilitates the function of NK cells by downregulating deubiquitinating enzyme CYLD expression (17). A similar experimental approach was used to analyze long ncRNA differences in NK cells from different tissues, and a novel long ncRNA, lnc-CD56, was identified, which positively regulates CD56 in human NK cells (18).

Although some progress has been made, research in this area is relatively scarce. More importantly, the transcriptional regulation program of NK cell development is a “cat’s cradle” of networks performing at multiple levels. Thus, as with any single-factor analysis, understanding the molecular program of NK cell development completely is challenging. “Multi-omics” can help (i) predict and analyze new regulatory elements and (ii) better understand the molecular mechanisms of transcriptional regulation in NK cell development (Figure 2).

## “Omics” Analysis Sheds Light on the Diversity of NK Cells

Traditionally, NK cells have been thought to be a homogenous population derived from the bone marrow and which circulate throughout peripheral tissues. In recent years, studies have shown that NK cells constitute various unique subsets with different phenotypes and functions (45, 75, 76).

From the perspective of NK cells in the liver, in 2013, Tian’s group is the first to identify CD49a^+^DX5^−^ NK cells as trNK cells. Through a comprehensive transcriptome obtained *via* microarray and fluorescence-activated cell sorting analyses (32, 77–80), they suggested that lineages of trNK cells in the liver may be different from cNK cells in the spleen. The discovery of liver trNK cells has rejuvenated scholars and will lead to trNK research in other areas (81). In 2014, Yokoyama’s group showed that CD49a^+^ trNK cells are present not only in the liver but also in the skin and uterine tissue (19). RNA-seq and multiple TF gene deficiencies in mice were used to provide more complete evidence to answer why trNK cells are different to cNK cells, especially in terms of TFs requirements. Their data confirmed the notion that the development of trNK cells in the liver is independent of GATA-3 or NFIL3 but dependent on T-bet (19). After that discovery, it was revealed that a “T-bet^+^ Eomes^−^ CD49a^+^ NK cell subsets” was present in the human liver (82). However, CD49a^+^ NK cells in the human liver may be present in variable quantities.

Cytometry by time of flight can be used to discover and define unique cell populations even if a specific marker for a given subset is not used. By using CyTOF and humanized mice, Yokoyama and colleagues showed that CD49e^−^ is a characteristic marker of trNK cells (25). In addition, several research teams have also reported that the trNK cells observed in the uterus, kidney (83), and salivary glands (84, 85) are different from cNK cells in terms of origin, development, and function using “-omics” analysis. Thanks to progress in two-photon microscopy, the discovery of extramedullary hematopoiesis in the liver, spleen (86), and even lungs (87) has better defined the origin of trNK cells.

Thanks to “-omics” analysis, the emergence of NK cell diversity based on tissue specificity or the production of different cytokines and the recently identified ILCs have led to a new nomenclature that assigns cNK cells into ILC1s (88, 89). Current studies on ILCs are dependent mainly on a mouse model, and technical limitations (e.g., multicolor FCM requires at least eight fluorescence channels; spectral overlap) have hampered adequate characterization of human ILCs. CyTOF provided considerable help to Simoni and colleagues in profiling ILCs from human tissues. Surprisingly, they showed that ILC1s (gating strategy: CD45^+^Lin^−^CD94^−^CD127^+^ CRTH2^−^c-Kit^−^NKp44^−^) were undetectable in human tissues, and an intraepithelial ILC1-like population not restricted to mucosal tissues and which displayed similarity to NK cells was found (90). Bernink et al. showed that NK cells could be distinguished from ILC1s because NK cells highly expressed EOMES, perforin, and granzyme B along with a lack of cell surface expression of CD127 and CD49a (91). A more interesting finding was that NK cells and ILC1s had more closely overlapping gene expression on phenotypes and functional programs (15). Although NK cells have similar functions to ILCs, they may be derived from distinct progenitors and have different requirements for EOMES and T-bet (92). Those studies seem to suggest that these two cell types represent only a subset of the broad NK lineage (93).

The emerging knowledge of the diversity of ILC2s and ILC3s is important (90, 93). The diversity of ILCs is a very complicated and confusing problem. A more optimized “multi-omics” analysis uncovered the veil of the diversity of NK cells and allowed us to better understand how cell diversity affects their functions in different tissues in physiologic and pathologic conditions (Figure 2).

## “Omics” Analysis Will Accelerate Research into NK Cells and Start a New Chapter in Immunotherapy

Natural killer cells spontaneously kill cells that are deemed to be “dangerous” to the host, including tumor cells (1) and viruses (94). NK cells have been valuable for fighting against cancer, and researchers are now close to a big breakthrough: NK cells may be able to identify and rapidly kill tumor cells without damaging healthy cells or risking the “storm” of pro-inflammatory cytokines caused by activated T cells (6).

Recently, due to the wider applications of “-omics” analysis, including scRNA-seq and CyTOF, the classifications and descriptions of NK cell subsets have reached a new level (95). Using CyTOF combined with analyses rooted in epidemiology and population genetics, it not only showed that haplotypes with −21M HLA-B rarely encode the KIR ligands Bw4^+^HLA-B and C2^+^HLA-C KIR but also showed that stepwise addition of each KIR ligand associated with NK cells helped to “educate” and recognize the responses of CD94:NKG2A and HLA-E (96, 97). In fact, those findings suggest new ways to dissect the numerous clinical associations with HLA class-I molecules and are important for the clinical application of NK cells (98). Romee and colleagues investigated the potential of memory-like NK cells in cancer therapy. Through CyTOF, SPADE analysis was used in memory-like NK cells pre-activated by IL-12, IL-15, and IL-18. Results showed that these cells were effective against leukemia targets regardless of KIR–KIR ligand interactions (48). In a study by Miller and colleagues in 2005, this new treatment strategy using pre-activation elicited greater progress than direct transfer of NK cells in inducing the remission of AML (99). As an “off-the-shelf” therapy, on 20 March 2017, the US Food and Drug Administration granted a designation of “orphan drug” to the NantKwest Company for activated NK cell therapy for patients diagnosed with malignant Merkel cell carcinoma. We believe that massive “-omics” data will provide more information to immunologists for developing more accurate and effective NK cell therapy for tumor immunotherapy.

“Omics” analysis is also widely used in antiviral studies using NK cells. Memory-like NK cells have been induced in viral-infected mice (100–102), but the formation mechanism of the pool of memory-like NK cells is not clear. Results of transcriptome and DNA methylation analyses have shown that the formation and maintenance of memory-like NK cells is dependent on the epigenetic changes associated with functional changes (103) and antibody-dependent expansion (104). Moreover, there is insufficient evidence for a correlation between the diversity and the function of NK cells. CyTOF has been used to assess changes in NK cell diversity during human immunodeficiency virus (HIV) infection. Results showed that an increase in NK cell diversity could reduce the ability of expansion and degranulation though promotion of the secretion of cytokines, which resulted in an increased risk of HIV infection (24). Recently, Aguilar et al. identified the viral ligand m12 for NK1.1 (105) receptors through protein structure-related big data analysis (106). That study has elicited considerable progress in the study of NK cells and has important implications for immunotherapy.

In addition, human cytomegalovirus (HCMV) infection has been shown to be related to some autoimmune diseases (ADs) (107–109) and regulatory NK cells (110–114). An HCMV-induced autoantibody was identified from AD patients using phage display technology and provided a clear intrinsic connection between reduced numbers of CD56^bright^ NK cells caused by autoantibodies and AD (115). In addition, during a successful pregnancy, NK cells act as crucial regulatory cells, producing IFN-γ to suppress Th17-mediated inflammation at the maternal–fetal interface (116). However, the regulation of NK cells has backfired in insulin resistance; experimental data show that stimulated NK cells are linked to obesity-induced adipose stress and lead to increased numbers of pro-inflammatory macrophages and exacerbate insulin resistance (117, 118). Irrespective of their use in the treatment of tumors, viral infections, or ADs, NK cells will usher in breakthroughs due to advancements in “-omics” technologies (Figure 2).

## Concluding Remarks

Natural killer cells are more complicated than originally thought. Due to technical limitations, for a long time, the study of NK cells lagged behind those of T cells and B cells. Reviewing the timeline of studies of NK cells, breakthroughs have been in parallel with advances in “-omics” technology. Such advances have not only been translated into new powerful tools but have also rejuvenated research into NK cells. “Omics” technology can provide an overwhelming amount of information in one experiment. Massive amounts of information can give immunologists richer clues and more ample data to better answer questions that remain regarding the biology of NK cells and further enhance understanding of NK cells (Figure 2). Moreover, “-omics” technology is a golden opportunity to accelerate the process of exploring the basic research of NK cells and developing NK cell-mediated immunotherapy to combat various diseases.

## Author Contributions

YZ collated data and wrote the review. XX collected data from online databases. ZT and HW conceived and edited the review.

## Conflict of Interest Statement

The authors declare that the research was conducted in the absence of any commercial or financial relationships that could be construed as a potential conflict of interest.
