# Supplementary material for: “Multi-Omics” Analyses of the Development and Function of Natural Killer Cells
Source: Front Immunol. 2017 Sep 5;8:1095. doi: 10.3389/fimmu.2017.01095 (PMC5591885; doi:10.3389/fimmu.2017.01095)
Supplement: Supplementary file 1 [file data_sheet_1.docx]

**Figure 1 refers to the reference. (In chronological order)**

1969, FACS: ([Hulett et al., 1969](#_ENREF_17))

1975, Natural cytotoxic cells: ([Kiessling et al., 1975](#_ENREF_18))

1977, 1-colour FCM using mAb: ([Williams et al., 1977](#_ENREF_36))

1979, IFN augment cytotoxicity and ADCC: ([Herberman et al., 1979](#_ENREF_15))

1979, Cytotoxicity by cultured human lymphocytes: ([Ortaldo et al., 1979](#_ENREF_25)).

1984, anti-NK-1.1 mAb, PK136: ([Koo and Peppard, 1984](#_ENREF_20))

1986, Deﬁnition of NK cells: ([Lanier et al., 1986](#_ENREF_21))

1988, PCR with thermostable Taq polymerase: ([Saiki et al., 1988](#_ENREF_28))

1989, Identity of CD56+ NK cells: ([Lanier et al., 1989](#_ENREF_22))

1991, Intracellular cytokine staining: ([Sander et al., 1991](#_ENREF_29))

1994, IL-15 activate NK cells by IL-2 receptor: ([Carson et al., 1994](#_ENREF_6))

1995, cDNA microarrays: ([Schena et al., 1995](#_ENREF_30))

1996, Real-time auantitative PCR: ([Heid et al., 1996](#_ENREF_14))

1997, CFSE in proliferation assay: ([Wells et al., 1997](#_ENREF_35))

1997, Identified specific marker of NK cells: NKp46: ([Sivori et al., 1997](#_ENREF_31))

2001, 11-colour FCM: ([De Rosa et al., 2001](#_ENREF_9))

2003, LC-MS/MS: ([Peng et al., 2003](#_ENREF_27))

2005, Successful adoptive of NK cell transfer to treat cancer: ([Miller et al., 2005](#_ENREF_23))

2005, Licensing of NK cell by MHC class I: ([Fernandez et al., 2005](#_ENREF_11)),([Kim et al., 2005](#_ENREF_19))

2006, NK cell education by MHC class I: ([Anfossi et al., 2006](#_ENREF_2))

2006, 18-colour FCM: ([Chattopadhyay et al., 2006](#_ENREF_7))

2007, ChIP-seq: ([Barski et al., 2007](#_ENREF_3))

2008, RNA-seq: ([Mortazavi et al., 2008](#_ENREF_24))

2008, Nanostring: ([Geiss et al., 2008](#_ENREF_13))

2009, Single cell RNA-seq: ([Tang et al., 2009](#_ENREF_34))

2009, Discovery of NK cell memory responses: ([Sun et al., 2009](#_ENREF_33))

2009, E4BP4 determine NK cell development: ([Gascoyne et al., 2009](#_ENREF_12))

2011, Over thirty markers mass cytometry (CyTOF): ([Bendall et al., 2011](#_ENREF_4))

2013, Revealed human NK cell diversity by CyTOF: ([Horowitz et al., 2013](#_ENREF_16))

2013, CD49^+^ liver resident NK cells: ([Peng et al., 2013](#_ENREF_26))

2013, Deﬁnition of ILC cells: ([Spits et al., 2013](#_ENREF_32))

2013, ATAC-seq: ([Buenrostro et al., 2013](#_ENREF_5))

2013, Gene editing mouse by CRISPR/cas9: ([Cong et al., 2013](#_ENREF_8))

2015, FOXO1 negatively regulates NK cell: ([Deng et al., 2015](#_ENREF_10))

2017, Identify a viral ligand for NK1.1, m12: ([Aguilar et al., 2017](#_ENREF_1))

Aguilar, O.A., Berry, R., Rahim, M.M., Reichel, J.J., Popovic, B., Tanaka, M., et al. (2017). A Viral Immunoevasin Controls Innate Immunity by Targeting the Prototypical Natural Killer Cell Receptor Family. *Cell* 169(1)**,** 58-71 e14. doi: 10.1016/j.cell.2017.03.002.

Anfossi, N., Andre, P., Guia, S., Falk, C.S., Roetynck, S., Stewart, C.A., et al. (2006). Human NK cell education by inhibitory receptors for MHC class I. *Immunity* 25(2)**,** 331-342. doi: 10.1016/j.immuni.2006.06.013.

Barski, A., Cuddapah, S., Cui, K., Roh, T.Y., Schones, D.E., Wang, Z., et al. (2007). High-resolution profiling of histone methylations in the human genome. *Cell* 129(4)**,** 823-837. doi: 10.1016/j.cell.2007.05.009.

Bendall, S.C., Simonds, E.F., Qiu, P., Amir el, A.D., Krutzik, P.O., Finck, R., et al. (2011). Single-cell mass cytometry of differential immune and drug responses across a human hematopoietic continuum. *Science* 332(6030)**,** 687-696. doi: 10.1126/science.1198704.

Buenrostro, J.D., Giresi, P.G., Zaba, L.C., Chang, H.Y., and Greenleaf, W.J. (2013). Transposition of native chromatin for fast and sensitive epigenomic profiling of open chromatin, DNA-binding proteins and nucleosome position. *Nat Methods* 10(12)**,** 1213-1218. doi: 10.1038/nmeth.2688.

Carson, W.E., Giri, J.G., Lindemann, M.J., Linett, M.L., Ahdieh, M., Paxton, R., et al. (1994). Interleukin (IL) 15 is a novel cytokine that activates human natural killer cells via components of the IL-2 receptor. *J Exp Med* 180(4)**,** 1395-1403.

Chattopadhyay, P.K., Price, D.A., Harper, T.F., Betts, M.R., Yu, J., Gostick, E., et al. (2006). Quantum dot semiconductor nanocrystals for immunophenotyping by polychromatic flow cytometry. *Nat Med* 12(8)**,** 972-977. doi: 10.1038/nm1371.

Cong, L., Ran, F.A., Cox, D., Lin, S., Barretto, R., Habib, N., et al. (2013). Multiplex genome engineering using CRISPR/Cas systems. *Science* 339(6121)**,** 819-823. doi: 10.1126/science.1231143.

De Rosa, S.C., Herzenberg, L.A., Herzenberg, L.A., and Roederer, M. (2001). 11-color, 13-parameter flow cytometry: identification of human naive T cells by phenotype, function, and T-cell receptor diversity. *Nat Med* 7(2)**,** 245-248. doi: 10.1038/84701.

Deng, Y., Kerdiles, Y., Chu, J., Yuan, S., Wang, Y., Chen, X., et al. (2015). Transcription factor Foxo1 is a negative regulator of natural killer cell maturation and function. *Immunity* 42(3)**,** 457-470. doi: 10.1016/j.immuni.2015.02.006.

Fernandez, N.C., Treiner, E., Vance, R.E., Jamieson, A.M., Lemieux, S., and Raulet, D.H. (2005). A subset of natural killer cells achieves self-tolerance without expressing inhibitory receptors specific for self-MHC molecules. *Blood* 105(11)**,** 4416-4423. doi: 10.1182/blood-2004-08-3156.

Gascoyne, D.M., Long, E., Veiga-Fernandes, H., de Boer, J., Williams, O., Seddon, B., et al. (2009). The basic leucine zipper transcription factor E4BP4 is essential for natural killer cell development. *Nat Immunol* 10(10)**,** 1118-1124. doi: 10.1038/ni.1787.

Geiss, G.K., Bumgarner, R.E., Birditt, B., Dahl, T., Dowidar, N., Dunaway, D.L., et al. (2008). Direct multiplexed measurement of gene expression with color-coded probe pairs. *Nat Biotechnol* 26(3)**,** 317-325. doi: 10.1038/nbt1385.

Heid, C.A., Stevens, J., Livak, K.J., and Williams, P.M. (1996). Real time quantitative PCR. *Genome Res* 6(10)**,** 986-994.

Herberman, R.R., Ortaldo, J.R., and Bonnard, G.D. (1979). Augmentation by interferon of human natural and antibody-dependent cell-mediated cytotoxicity. *Nature* 277(5693)**,** 221-223.

Horowitz, A., Strauss-Albee, D.M., Leipold, M., Kubo, J., Nemat-Gorgani, N., Dogan, O.C., et al. (2013). Genetic and environmental determinants of human NK cell diversity revealed by mass cytometry. *Sci Transl Med* 5(208)**,** 208ra145. doi: 10.1126/scitranslmed.3006702.

Hulett, H.R., Bonner, W.A., Barrett, J., and Herzenberg, L.A. (1969). Cell sorting: automated separation of mammalian cells as a function of intracellular fluorescence. *Science* 166(3906)**,** 747-749.

Kiessling, R., Klein, E., and Wigzell, H. (1975). "Natural" killer cells in the mouse. I. Cytotoxic cells with specificity for mouse Moloney leukemia cells. Specificity and distribution according to genotype. *Eur J Immunol* 5(2)**,** 112-117. doi: 10.1002/eji.1830050208.

Kim, S., Poursine-Laurent, J., Truscott, S.M., Lybarger, L., Song, Y.J., Yang, L., et al. (2005). Licensing of natural killer cells by host major histocompatibility complex class I molecules. *Nature* 436(7051)**,** 709-713. doi: 10.1038/nature03847.

Koo, G.C., and Peppard, J.R. (1984). Establishment of monoclonal anti-Nk-1.1 antibody. *Hybridoma* 3(3)**,** 301-303. doi: 10.1089/hyb.1984.3.301.

Lanier, L.L., Phillips, J.H., Hackett, J., Jr., Tutt, M., and Kumar, V. (1986). Natural killer cells: definition of a cell type rather than a function. *J Immunol* 137(9)**,** 2735-2739.

Lanier, L.L., Testi, R., Bindl, J., and Phillips, J.H. (1989). Identity of Leu-19 (CD56) leukocyte differentiation antigen and neural cell adhesion molecule. *J Exp Med* 169(6)**,** 2233-2238.

Miller, J.S., Soignier, Y., Panoskaltsis-Mortari, A., McNearney, S.A., Yun, G.H., Fautsch, S.K., et al. (2005). Successful adoptive transfer and in vivo expansion of human haploidentical NK cells in patients with cancer. *Blood* 105(8)**,** 3051-3057. doi: 10.1182/blood-2004-07-2974.

Mortazavi, A., Williams, B.A., McCue, K., Schaeffer, L., and Wold, B. (2008). Mapping and quantifying mammalian transcriptomes by RNA-Seq. *Nat Methods* 5(7)**,** 621-628. doi: 10.1038/nmeth.1226.

Ortaldo, J.R., Bonnard, G.D., Kind, P.D., and Herberman, R.B. (1979). Cytotoxicity by cultured human lymphocytes: characteristics of effector cells and specificity of cytotoxicity. *J Immunol* 122(4)**,** 1489-1494.

Peng, H., Jiang, X., Chen, Y., Sojka, D.K., Wei, H., Gao, X., et al. (2013). Liver-resident NK cells confer adaptive immunity in skin-contact inflammation. *J Clin Invest* 123(4)**,** 1444-1456. doi: 10.1172/jci66381.

Peng, J., Elias, J.E., Thoreen, C.C., Licklider, L.J., and Gygi, S.P. (2003). Evaluation of multidimensional chromatography coupled with tandem mass spectrometry (LC/LC-MS/MS) for large-scale protein analysis: the yeast proteome. *J Proteome Res* 2(1)**,** 43-50.

Saiki, R.K., Gelfand, D.H., Stoffel, S., Scharf, S.J., Higuchi, R., Horn, G.T., et al. (1988). Primer-directed enzymatic amplification of DNA with a thermostable DNA polymerase. *Science* 239(4839)**,** 487-491.

Sander, B., Andersson, J., and Andersson, U. (1991). Assessment of cytokines by immunofluorescence and the paraformaldehyde-saponin procedure. *Immunol Rev* 119**,** 65-93.

Schena, M., Shalon, D., Davis, R.W., and Brown, P.O. (1995). Quantitative monitoring of gene expression patterns with a complementary DNA microarray. *Science* 270(5235)**,** 467-470.

Sivori, S., Vitale, M., Morelli, L., Sanseverino, L., Augugliaro, R., Bottino, C., et al. (1997). p46, a novel natural killer cell-specific surface molecule that mediates cell activation. *J Exp Med* 186(7)**,** 1129-1136.

Spits, H., Artis, D., Colonna, M., Diefenbach, A., Di Santo, J.P., Eberl, G., et al. (2013). Innate lymphoid cells--a proposal for uniform nomenclature. *Nat Rev Immunol* 13(2)**,** 145-149. doi: 10.1038/nri3365.

Sun, J.C., Beilke, J.N., and Lanier, L.L. (2009). Adaptive immune features of natural killer cells. *Nature* 457(7229)**,** 557-561. doi: 10.1038/nature07665.

Tang, F., Barbacioru, C., Wang, Y., Nordman, E., Lee, C., Xu, N., et al. (2009). mRNA-Seq whole-transcriptome analysis of a single cell. *Nat Methods* 6(5)**,** 377-382. doi: 10.1038/nmeth.1315.

Wells, A.D., Gudmundsdottir, H., and Turka, L.A. (1997). Following the fate of individual T cells throughout activation and clonal expansion. Signals from T cell receptor and CD28 differentially regulate the induction and duration of a proliferative response. *J Clin Invest* 100(12)**,** 3173-3183. doi: 10.1172/jci119873.

Williams, A.F., Galfre, G., and Milstein, C. (1977). Analysis of cell surfaces by xenogeneic myeloma-hybrid antibodies: differentiation antigens of rat lymphocytes. *Cell* 12(3)**,** 663-673.
